# Water quality data in a shallow and narrow Setiu Lagoon

**DOI:** 10.1016/j.dib.2021.106866

**Published:** 2021-02-11

**Authors:** Zuraini Zainol, Mohd Fadzil Akhir, Afifi Johari, Azizi Ali

**Affiliations:** Institute of Oceanography and Environment, Universiti Malaysia Terengganu, 21030 Kuala Nerus, Terengganu, Malaysia

**Keywords:** Lagoon, Temperature, Salinity, Chlorophyll-*a*, Nutrients, Anthropogenic activities

## Abstract

This article contains water quality data collected in a shallow and narrow Setiu Lagoon during the southwest monsoon, wet period of northeast monsoon and dry period of northeast monsoon. The surface water quality parameters, which include the temperature, salinity, chlorophyll-*a* and nutrients (ammonia, nitrate, phosphate, and silicate) were sampled twice per day (high and low tides) at a total of eight stations. Hourly current speed and direction was obtained from mooring of two units of current meters. Compared to the Malaysia Marine Water Quality Criteria and Standard (MWQCS), nutrients in Setiu Lagoon were in Class 2. Although limited, this dataset can provide insights on the changes of water quality condition in Setiu Lagoon under the presence of anthropogenic pressures.

## Specifications Table

**Table:** 

Subject	Environmental Science
Specific subject area	Ecology and Pollution
Type of data	Table and Figure
How data were acquired	Using Sontek Castaway conductivity, temperature, and depth (CTD) profiler for *in situ* measurements of temperature, salinity and depth; using Westco Smartchem discrete analyser for inorganic nutrients; using UV–VIS spectrophotometer for chlorophyll-*a* and using Valeport current meter for current speed and direction.
Data format	Raw and analysed
Parameters for data collection	For inorganic nutrients: Water samples were filtered on-site by 0.7 µm GF/F filter paper and kept frozen until further analysis. For chlorophyll-*a*: Filter papers were rolled, kept in 15 ml centrifuge tubes and stored frozen until further analysis.
Description of data collection	Water quality data were collected from eight stations in Setiu Lagoon. Water samples were collected for inorganic nutrients and chlorophyll-*a* analyses. Current speed and direction were measured at two mooring points in Setiu Lagoon.
Data source location	Water quality data were collected in Setiu Lagoon, which is situated in Setiu Wetland, Terengganu, Malaysia.
Data accessibility	With the article
Related research article	Zainol, Z., Akhir, M. F., and Abdullah, S. (2020). Hydrodynamics, nutrient concentrations, and phytoplankton biomass in a shallow and restricted coastal lagoon under different tidal and monsoonal environmental drivers. Regional Studies in Marine Science, 38, 101,376. https://doi.org/10.1016/j.rsma.2020.101376

## Value of the Data

• The data reflects the current status of water quality in Setiu Lagoon that could be used to investigate the changes of parameters in relation to different tidal and monsoonal forces.
• Although limited, the data could be helpful in monitoring the water quality condition of Setiu Lagoon with the presence of aquaculture and agriculture activities within vicinity, which may be of interest to scientists, responsible authorities and local residents.
• The comparison made between the water quality data against Malaysia Marine Water Quality Criteria and Standard (MWQCS), could be a great tool in delivering the information of the current status of this area to the policy maker.
• The hourly current speed and direction data, which is still sparse in Setiu Lagoon, would be a great help to scientists that keen to understand the hydrodynamics of this area and its role in affecting the distribution of biogeochemical parameters.
• The findings could be applied to other estuary and lagoon ecosystems with similar shallow and narrow features, in which limited capability for water renewal due to the restricted characteristics might put a great pressure on the water quality on account of anthropogenic factors.

## 1 Data Description

**Fig. 1:**
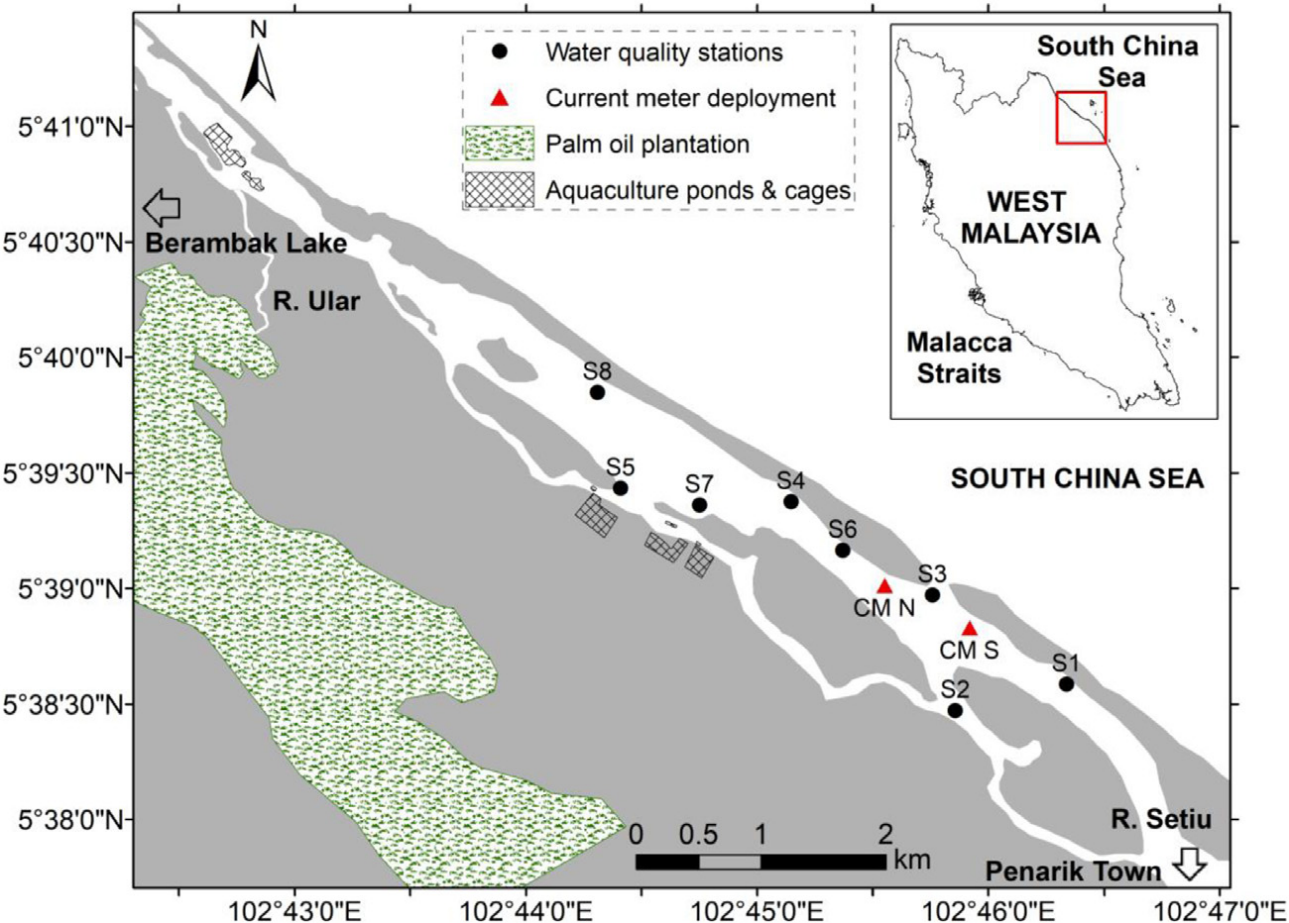
Map showing location of sampling stations in Setiu Lagoon modified from reference [1].

Water quality data provided in this article were collected at a total of eight stations (namely S1 to S8), which were randomly distributed in Setiu Lagoon, Terengganu, Malaysia during August 2017, December 2017 and February 2018 (Table 1). The date and time in Table 1 represents the sampling period. Locations and descriptions of each station were provided in Fig. 1 and Table 2, while summary of the sampling duration was tabulated in Table 3. Table 4 displays the hourly current speed and direction data during the sampling period, which was obtained from the moored current meters at CM N and CM S stations (Fig. 1). The water quality data collected during the high and low tides were used to calculate the mean and standard deviation, while the hourly current speed and direction were used to estimate the flow rate in the journal publication (reference number 1). Further, the mean nutrient concentrations were also compared with the Malaysia Marine Water Quality Criteria and Standard (MWQCS) in assessing the current status of the area (Table 5).

**Table 1:** Water quality measurements in Setiu Lagoon during August 2017, December 2017 and February 2018.

Date	Time	Tidal	St	Temp (°C)	Salinity (psu)	Chl-*a* (µg/L)	NH_3_ (µM)	NO_3_ (µM)	PO_4_ (µM)	SiO_4_ (µM)
7/8/2017	9:20:00	HT	S1	30.48	32.55	0.30	1.63	1.46	0.08	16.01
7/8/2017	9:25:00	HT	S2	30.45	32.58	0.71	4.36	1.39	0.15	20.00
7/8/2017	9:35:00	HT	S3	30.45	32.62	0.75	1.63	0.54	0.18	11.12
7/8/2017	9:45:00	HT	S4	30.61	31.93	0.57	2.71	1.10	0.16	48.93
7/8/2017	9:55:00	HT	S5	30.66	32.37	0.54	2.30	0.51	0.11	21.30
7/8/2017	9:39:00	HT	S6	30.69	32.34	–	–	–	–	–
7/8/2017	15:52:00	LT	S1	31.62	23.50	0.65	7.20	4.20	0.04	98.71
7/8/2017	16:13:00	LT	S2	31.99	24.46	0.26	7.61	2.46	0.11	73.87
7/8/2017	15:29:00	LT	S3	31.87	30.37	0.99	3.27	0.89	0.09	62.50
7/8/2017	15:19:00	LT	S4	32.18	30.47	1.10	2.01	0.76	0.09	48.13
7/8/2017	15:12:00	LT	S5	31.61	23.75	0.88	8.34	2.68	0.23	76.86
7/8/2017	15:25:00	LT	S6	32.16	30.66	–	–	–	–	–
8/8/2017	10:24:00	HT	S1	30.88	32.20	0.83	1.80	0.52	0.12	12.42
8/8/2017	10:33:00	HT	S2	30.65	32.41	0.56	3.72	0.38	0.09	16.51
8/8/2017	10:14:00	HT	S3	30.55	32.42	1.46	2.63	2.08	0.06	60.90
8/8/2017	10:05:00	HT	S4	30.87	32.18	1.05	1.88	0.52	0.12	20.70
8/8/2017	9:57:00	HT	S5	30.82	28.61	1.66	5.05	1.57	0.10	47.23
8/8/2017	10:09:00	HT	S6	30.79	32.28	–	–	–	–	–
8/8/2017	15:35:00	LT	S1	32.01	28.76	1.32	4.55	1.56	0.11	42.15
8/8/2017	15:50:00	LT	S2	31.74	28.01	0.87	4.48	1.80	0.16	61.90
8/8/2017	15:21:00	LT	S3	32.03	30.39	1.45	3.37	1.18	0.12	40.55
8/8/2017	15:12:00	LT	S4	32.34	31.29	0.82	1.88	1.14	0.12	38.36
8/8/2017	15:23:00	LT	S5	31.87	25.98	2.53	4.86	3.19	0.18	84.04
8/8/2017	15:17:00	LT	S6	32.22	31.80	–	–	–	–	–
27/12/2017	15:48:00	LT	S1	26.78	1.37	0.27	8.92	10.02	0.25	110.86
27/12/2017	16:05:00	LT	S2	26.84	6.64	0.19	4.30	3.33	0.26	64.26
27/12/2017	16:00:00	LT	S3	27.03	7.07	0.19	4.22	–	0.31	103.70
27/12/2017	15:20:00	LT	S4	27.99	17.96	1.31	2.97	3.25	0.18	65.82
27/12/2017	14:54:00	LT	S5	27.48	12.93	0.45	4.55	3.24	0.18	83.20
27/12/2017	15:27:00	LT	S6	27.50	16.19	1.28	3.78	2.97	0.11	85.95
27/12/2017	15:13:00	LT	S7	27.44	14.83	0.95	3.99	2.29	0.11	74.20
27/12/2017	15:00:00	LT	S8	27.86	15.43	1.10	3.57	3.10	0.14	73.68
27/12/2017	9:25:00	HT	S1	27.30	0.42	0.77	5.97	3.13	0.05	28.27
27/12/2017	10:10:00	HT	S2	27.32	2.64	0.53	4.18	2.00	0.15	52.52
27/12/2017	9:39:00	HT	S3	27.33	0.47	0.45	4.64	2.77	0.07	19.27
27/12/2017	9:22:00	HT	S4	28.57	15.72	3.77	1.73	1.73	0.09	37.84
27/12/2017	8:47:00	HT	S5	28.47	10.59	1.32	4.19	1.74	0.11	66.81
27/12/2017	9:28:00	HT	S6	28.15	9.31	1.37	2.63	1.32	0.06	44.04
27/12/2017	9:16:00	HT	S7	28.66	15.47	3.85	2.34	2.63	0.12	76.43
27/12/2017	9:00:00	HT	S8	28.54	14.10	3.24	3.13	3.36	0.24	104.84
28/12/2017	9:22:00	LT	S1	26.54	3.87	0.40	5.05	–	0.37	219.75
28/12/2017	9:38:00	LT	S2	26.60	5.88	0.58	5.17	6.02	0.43	206.97
28/12/2017	9:10:00	LT	S3	26.77	8.32	0.29	5.32	5.29	0.34	195.93
28/12/2017	8:54:00	LT	S4	27.79	15.95	1.45	3.43	3.18	0.26	89.80
28/12/2017	8:25:00	LT	S5	27.63	13.59	0.81	4.89	3.72	0.31	142.31
28/12/2017	9:00:00	LT	S6	27.39	15.05	1.09	3.83	2.51	0.19	113.47
28/12/2017	8:48:00	LT	S7	27.52	13.87	1.27	4.94	3.46	0.43	130.73
28/12/2017	8:32:00	LT	S8	27.56	12.79	1.19	4.18	3.84	0.21	140.77
28/12/2017	15:42:00	HT	S1	27.89	0.36	1.68	8.47	6.91	0.28	92.74
28/12/2017	15:54:00	HT	S2	28.18	3.12	1.46	5.57	7.16	0.39	264.84
28/12/2017	15:31:00	HT	S3	28.44	28.00	0.86	2.40	1.69	0.18	44.57
28/12/2017	15:17:00	HT	S4	28.23	6.33	1.51	5.12	5.17	0.33	203.02
28/12/2017	14:50:00	HT	S5	29.25	10.68	1.88	4.53	3.07	0.23	153.78
28/12/2017	15:23:00	HT	S6	28.30	8.64	1.22	5.17	5.70	0.27	205.24
28/12/2017	15:09:00	HT	S7	29.03	11.49	6.73	2.66	4.03	0.24	149.78
28/12/2017	14:57:00	HT	S8	29.09	11.84	5.78	1.71	4.03	0.20	134.02
12/2/2018	10:55:00	LT	S1	28.06	6.66	0.34	3.90	6.02	0.77	247.15
12/2/2018	10:39:00	LT	S3	28.40	16.05	0.90	2.78	4.20	0.28	148.78
12/2/2018	10:16:00	LT	S4	28.73	21.67	2.18	1.05	2.43	0.15	63.67
12/2/2018	9:21:00	LT	S5	27.74	20.68	1.86	1.27	1.14	0.15	71.09
12/2/2018	10:25:00	LT	S6	28.67	21.91	2.88	1.03	1.27	0.13	58.75
12/2/2018	10:00:00	LT	S7	28.20	23.13	1.66	1.37	1.24	0.25	57.83
12/2/2018	9:35:00	LT	S8	28.89	18.74	3.01	0.57	1.35	0.41	83.15
12/2/2018	16:03:00	HT	S1	29.98	28.86	1.79	0.64	2.53	0.09	37.10
12/2/2018	16:23:00	HT	S2	30.77	26.22	1.96	1.69	2.12	0.18	76.88
12/2/2018	15:55:00	HT	S3	29.17	30.17	1.64	0.51	0.88	0.05	25.29
12/2/2018	15:40:00	HT	S4	30.03	24.31	3.40	0.49	0.46	0.14	56.53
12/2/2018	15:10:00	HT	S5	31.62	18.52	5.90	0.46	0.34	0.15	69.40
12/2/2018	15:45:00	HT	S6	30.06	26.56	1.70	0.67	1.26	0.14	57.26
12/2/2018	15:32:00	HT	S7	30.94	20.86	2.29	0.92	0.71	0.28	71.09
12/2/2018	15:18:00	HT	S8	31.03	18.42	3.43	0.69	0.35	0.21	60.20
13/2/2018	10:10:00	LT	S1	27.91	13.45	0.43	2.51	10.75	0.64	207.51
13/2/2018	9:57:00	LT	S3	28.25	27.69	0.81	1.18	2.45	0.29	79.34
13/2/2018	9:41:00	LT	S4	28.40	25.99	1.73	1.14	0.91	0.29	48.24
13/2/2018	9:15:00	LT	S5	28.53	25.98	1.37	1.52	1.13	0.11	75.48
13/2/2018	9:46:00	LT	S6	28.30	27.00	1.55	1.04	0.89	0.19	55.91
13/2/2018	9:35:00	LT	S7	28.26	27.02	0.85	1.17	0.96	0.21	49.88
13/2/2018	9:22:00	LT	S8	28.54	21.90	2.17	1.52	0.88	0.38	64.54
13/2/2018	15:11:00	HT	S1	29.71	21.01	0.99	3.06	4.41	0.42	212.14
13/2/2018	15:22:00	HT	S2	29.54	23.04	0.87	2.25	2.53	0.34	134.31
13/2/2018	15:00:00	HT	S3	29.27	23.25	2.60	0.20	0.38	0.15	23.45
13/2/2018	15:52:00	HT	S4	29.30	24.67	2.95	0.43	1.76	0.15	78.18
13/2/2018	16:14:00	HT	S5	28.70	29.86	2.62	0.50	0.69	0.15	62.27
13/2/2018	15:47:00	HT	S6	29.36	22.34	2.95	0.34	1.56	0.13	72.01
13/2/2018	15:58:00	HT	S7	28.94	17.94	3.73	0.60	0.86	0.50	75.24
13/2/2018	16:08:00	HT	S8	28.72	10.61	6.08	0.44	0.07	0.13	60.77
St = Station			Temp = Temperature				Chl-*a* = Chlorophyll-*a*			
NH_3_ = Dissolved ammonia			NO_3_ = Dissolved nitrate				PO_4_ = Dissolved phosphate			
SiO_4_ = Dissolved silicate

**Table 2:** Coordinates and descriptions of the sampling stations.

Station	Latitude (°N)	Longitude (°E)	Notes
S1	5.643073	102.772323	Downstream of Setiu River
S2	5.641194	102.764275	Downstream of Setiu River
S3	5.649486	102.762614	Inlet
S4	5.656241	102.752478	Lagoon area
S5	5.657233	102.740162	Located near to aquaculture site
S6	5.652717	102.756196	Lagoon area
S7	5.656017	102.745844	Located near to aquaculture site
S8	5.664111	102.738522	Lagoon area
CM N	5.650189	102.759167	Current meter deployment in the north of the inlet
CM S	5.647139	102.765290	Current meter deployment in the south of the inlet

**Table 3:** Descriptions of the sampling duration.

	High water (HW)		Low water (LW)		Measurement time	
Sampling date	Time	Tidal heights (m)	Time	Tidal heights (m)	HW	LW
7 Aug 2017	0824	1.7	1546	0.8	0910–0955	1512–1613
8 Aug 2017	0857	1.7	1555	0.8	0942–1033	1504–1550
27 Dec 2017	1443	1.4	0953	1.1	1500–1605	0847–1010
28 Dec 2017	1310	1.5	1010	1.1	1450–1540	0825–0938
12 Feb 2018	1510	1.8	1036	0.7	1510–1603	0921–1055
13 Feb 2018	1543	1.9	1134	0.6	1511–1614	0915–1010

**Table 4:** Current speed and direction in Setiu Lagoon during August 2017, December 2017 and February 2018.

Date	Time	St	CS (m/s)	CD (°)	Date	Time	ST	CS (m/s)	CD (°)
6/8/2017	12:04:43	CM N	0.43	71.5	6/8/2017	12:09:09	CM S	0.48	322.3
6/8/2017	13:04:43	CM N	0.54	69.8	6/8/2017	13:09:09	CM S	0.59	322.4
6/8/2017	14:04:43	CM N	0.61	66.8	6/8/2017	14:09:09	CM S	0.67	320.5
6/8/2017	15:04:43	CM N	0.59	63.6	6/8/2017	15:09:09	CM S	0.60	322.1
6/8/2017	16:04:43	CM N	0.55	62.5	6/8/2017	16:09:09	CM S	0.54	320.9
6/8/2017	17:04:43	CM N	0.40	63.8	6/8/2017	17:09:09	CM S	0.36	320.3
6/8/2017	18:04:43	CM N	0.34	65.1	6/8/2017	18:09:09	CM S	0.30	322.8
6/8/2017	19:04:43	CM N	0.37	60.9	6/8/2017	19:09:09	CM S	0.28	322.0
6/8/2017	20:04:43	CM N	0.53	62.2	6/8/2017	20:09:09	CM S	0.38	324.7
6/8/2017	21:04:43	CM N	0.55	59.2	6/8/2017	21:09:09	CM S	0.38	326.3
6/8/2017	22:04:43	CM N	0.58	56.6	6/8/2017	22:09:09	CM S	0.44	325.8
6/8/2017	23:04:43	CM N	0.57	56.3	6/8/2017	23:09:09	CM S	0.44	326.8
7/8/2017	0:04:43	CM N	0.50	66.2	7/8/2017	0:09:09	CM S	0.30	326.5
7/8/2017	1:04:43	CM N	0.25	53.9	7/8/2017	1:09:09	CM S	0.22	325.8
7/8/2017	2:04:43	CM N	0.24	53.4	7/8/2017	2:09:09	CM S	0.23	322.8
7/8/2017	3:04:43	CM N	0.05	47.0	7/8/2017	3:09:09	CM S	0.12	332.0
7/8/2017	4:04:43	CM N	0.29	236.2	7/8/2017	4:09:09	CM S	0.29	168.9
7/8/2017	5:04:43	CM N	0.52	246.6	7/8/2017	5:09:09	CM S	0.58	151.7
7/8/2017	6:04:43	CM N	0.63	255.0	7/8/2017	6:09:09	CM S	0.74	151.3
7/8/2017	7:04:43	CM N	0.61	261.0	7/8/2017	7:09:09	CM S	0.73	149.4
7/8/2017	8:04:43	CM N	0.56	266.8	7/8/2017	8:09:09	CM S	0.72	145.0
7/8/2017	9:04:43	CM N	0.51	271.8	7/8/2017	9:09:09	CM S	0.59	141.9
7/8/2017	10:04:43	CM N	0.37	275.3	7/8/2017	10:09:09	CM S	0.43	139.9
7/8/2017	11:04:43	CM N	0.03	314.3	7/8/2017	11:09:09	CM S	0.05	145.5
7/8/2017	12:04:43	CM N	0.35	73.8	7/8/2017	12:09:09	CM S	0.36	324.0
7/8/2017	13:04:43	CM N	0.52	71.6	7/8/2017	13:09:09	CM S	0.56	322.8
7/8/2017	14:04:43	CM N	0.65	67.3	7/8/2017	14:09:09	CM S	0.72	322.0
7/8/2017	15:04:43	CM N	0.67	64.0	7/8/2017	15:09:09	CM S	0.70	321.2
7/8/2017	16:04:43	CM N	0.62	62.6	7/8/2017	16:09:09	CM S	0.61	323.8
7/8/2017	17:04:43	CM N	0.50	61.5	7/8/2017	17:09:09	CM S	0.49	323.6
7/8/2017	18:04:43	CM N	0.40	62.4	7/8/2017	18:09:09	CM S	0.38	325.5
7/8/2017	19:04:43	CM N	0.27	61.8	7/8/2017	19:09:09	CM S	0.22	327.8
7/8/2017	20:04:43	CM N	0.28	61.3	7/8/2017	20:09:09	CM S	0.25	327.9
7/8/2017	21:04:43	CM N	0.49	59.0	7/8/2017	21:09:09	CM S	0.38	329.2
7/8/2017	22:04:43	CM N	0.59	58.5	7/8/2017	22:09:09	CM S	0.45	328.5
7/8/2017	23:04:43	CM N	0.65	55.0	7/8/2017	23:09:09	CM S	0.44	328.2
8/8/2017	0:04:43	CM N	0.57	55.9	8/8/2017	0:09:09	CM S	0.48	329.9
8/8/2017	1:04:43	CM N	0.49	57.4	8/8/2017	1:09:09	CM S	0.34	330.9
8/8/2017	2:04:43	CM N	0.36	59.4	8/8/2017	2:09:09	CM S	0.24	332.7
8/8/2017	3:04:43	CM N	0.00	60.0	8/8/2017	3:09:09	CM S	0.15	336.5
8/8/2017	4:04:43	CM N	0.00	233.1	8/8/2017	4:09:09	CM S	0.01	156.6
8/8/2017	5:04:43	CM N	0.36	232.2	8/8/2017	5:09:09	CM S	0.57	160.3
8/8/2017	6:04:43	CM N	0.51	249.4	8/8/2017	6:09:09	CM S	0.64	152.1
8/8/2017	7:04:43	CM N	0.59	257.2	8/8/2017	7:09:09	CM S	0.78	150.3
8/8/2017	8:04:43	CM N	0.63	264.1	8/8/2017	8:09:09	CM S	0.78	148.6
8/8/2017	9:04:43	CM N	0.57	269.0	8/8/2017	9:09:09	CM S	0.72	144.2
8/8/2017	10:04:43	CM N	0.51	273.6	8/8/2017	10:09:09	CM S	0.57	140.5
8/8/2017	11:04:43	CM N	0.21	277.9	26/12/2017	13:09:48	CM S	0.41	319.7
26/12/2017	13:12:22	CM N	0.17	70.2	26/12/2017	14:09:48	CM S	0.50	317.2
26/12/2017	14:12:22	CM N	0.25	67.2	26/12/2017	15:09:48	CM S	0.48	316.1
26/12/2017	15:12:22	CM N	0.23	68.9	26/12/2017	16:09:48	CM S	0.38	316.7
26/12/2017	16:12:22	CM N	0.11	61.8	26/12/2017	17:09:48	CM S	0.37	317.6
26/12/2017	17:12:22	CM N	0.10	66.5	26/12/2017	18:09:48	CM S	0.27	317.6
26/12/2017	18:12:22	CM N	0.01	103.0	26/12/2017	19:09:48	CM S	0.26	317.9
26/12/2017	19:12:22	CM N	0.01	83.7	26/12/2017	20:09:48	CM S	0.13	316.0
26/12/2017	20:12:22	CM N	0.01	313.8	26/12/2017	21:09:48	CM S	0.02	329.8
26/12/2017	21:12:22	CM N	0.25	253.4	26/12/2017	22:09:48	CM S	0.35	135.4
26/12/2017	22:12:22	CM N	0.23	270.0	26/12/2017	23:09:48	CM S	0.28	136.2
26/12/2017	23:12:22	CM N	0.22	273.4	27/12/2017	0:09:48	CM S	0.28	136.1
27/12/2017	0:12:22	CM N	0.24	275.9	27/12/2017	1:09:48	CM S	0.27	135.1
27/12/2017	1:12:22	CM N	0.21	279.7	27/12/2017	2:09:48	CM S	0.18	134.0
27/12/2017	2:12:22	CM N	0.11	282.4	27/12/2017	3:09:48	CM S	0.04	142.0
27/12/2017	3:12:22	CM N	0.02	353.2	27/12/2017	4:09:48	CM S	0.15	317.9
27/12/2017	4:12:22	CM N	0.23	78.6	27/12/2017	5:09:48	CM S	0.36	315.6
27/12/2017	5:12:22	CM N	0.42	73.5	27/12/2017	6:09:48	CM S	0.37	312.0
27/12/2017	6:12:22	CM N	0.38	67.7	27/12/2017	7:09:48	CM S	0.52	311.3
27/12/2017	7:12:22	CM N	0.46	65.6	27/12/2017	8:09:48	CM S	0.56	320.0
27/12/2017	8:12:22	CM N	0.46	67.7	27/12/2017	9:09:48	CM S	0.49	319.4
27/12/2017	9:12:22	CM N	0.46	66.8	27/12/2017	10:09:48	CM S	0.48	317.1
27/12/2017	10:12:22	CM N	0.44	63.6	27/12/2017	11:09:48	CM S	0.46	319.3
27/12/2017	11:12:22	CM N	0.33	63.0	27/12/2017	12:09:48	CM S	0.38	318.3
27/12/2017	12:12:22	CM N	0.21	64.5	27/12/2017	13:09:48	CM S	0.39	316.3
27/12/2017	13:12:22	CM N	0.11	73.0	27/12/2017	14:09:48	CM S	0.28	316.5
27/12/2017	14:12:22	CM N	0.10	58.8	27/12/2017	15:09:48	CM S	0.14	315.3
27/12/2017	15:12:22	CM N	0.01	53.4	27/12/2017	16:09:48	CM S	0.21	318.5
27/12/2017	16:12:22	CM N	0.08	69.7	27/12/2017	17:09:48	CM S	0.15	317.1
27/12/2017	17:12:22	CM N	0.06	70.2	27/12/2017	18:09:48	CM S	0.18	317.5
27/12/2017	18:12:22	CM N	0.08	56.3	27/12/2017	19:09:48	CM S	0.13	314.7
27/12/2017	19:12:22	CM N	0.00	53.3	27/12/2017	20:09:48	CM S	0.10	319.0
27/12/2017	20:12:22	CM N	0.01	46.0	27/12/2017	21:09:48	CM S	0.15	124.0
27/12/2017	21:12:22	CM N	0.25	264.4	27/12/2017	22:09:48	CM S	0.20	132.3
27/12/2017	22:12:22	CM N	0.19	270.1	27/12/2017	23:09:48	CM S	0.30	137.4
27/12/2017	23:12:22	CM N	0.20	271.8	28/12/2017	0:09:48	CM S	0.29	137.1
28/12/2017	0:12:22	CM N	0.20	274.2	28/12/2017	1:09:48	CM S	0.30	135.5
28/12/2017	1:12:22	CM N	0.21	277.0	28/12/2017	2:09:48	CM S	0.22	135.1
28/12/2017	2:12:22	CM N	0.14	282.3	28/12/2017	3:09:48	CM S	0.14	139.2
28/12/2017	3:12:22	CM N	0.07	285.2	28/12/2017	4:09:48	CM S	0.13	314.7
28/12/2017	4:12:22	CM N	0.20	84.5	28/12/2017	5:09:48	CM S	0.27	318.9
28/12/2017	5:12:22	CM N	0.31	77.6	28/12/2017	6:09:48	CM S	0.38	312.0
28/12/2017	6:12:22	CM N	0.39	69.3	28/12/2017	7:09:48	CM S	0.49	313.9
28/12/2017	7:12:22	CM N	0.45	65.4	28/12/2017	8:09:48	CM S	0.61	315.1
28/12/2017	8:12:22	CM N	0.47	66.3	28/12/2017	9:09:48	CM S	0.55	318.5
28/12/2017	9:12:22	CM N	0.46	66.7	28/12/2017	10:09:48	CM S	0.55	317.8
28/12/2017	10:12:22	CM N	0.45	64.3	28/12/2017	11:09:48	CM S	0.46	321.3
28/12/2017	11:12:22	CM N	0.39	62.9	28/12/2017	12:09:48	CM S	0.26	322.2
28/12/2017	12:12:22	CM N	0.18	69.5	28/12/2017	13:09:48	CM S	0.19	324.9
28/12/2017	13:12:22	CM N	0.11	69.6	28/12/2017	14:09:48	CM S	0.07	324.7
28/12/2017	14:12:22	CM N	0.02	233.2	28/12/2017	15:09:48	CM S	0.06	105.7
28/12/2017	15:12:22	CM N	0.24	259.2	28/12/2017	16:09:48	CM S	0.23	137.5
28/12/2017	16:12:22	CM N	0.19	264.8	11/2/2018	13:02:33	CM S	0.01	322.8
11/2/2018	13:03:05	CM N	0.04	250.0	11/2/2018	14:02:33	CM S	0.25	136.7
11/2/2018	14:03:05	CM N	0.21	243.6	11/2/2018	15:02:33	CM S	0.44	133.4
11/2/2018	15:03:05	CM N	0.15	259.3	11/2/2018	16:02:33	CM S	0.61	135.7
11/2/2018	16:03:05	CM N	0.17	260.3	11/2/2018	17:02:33	CM S	0.58	137.2
11/2/2018	17:03:05	CM N	0.17	262.5	11/2/2018	18:02:33	CM S	0.56	137.3
11/2/2018	18:03:05	CM N	0.18	267.9	11/2/2018	19:02:33	CM S	0.48	139.1
11/2/2018	19:03:05	CM N	0.20	271.7	11/2/2018	20:02:33	CM S	0.33	138.2
11/2/2018	20:03:05	CM N	0.17	274.7	11/2/2018	21:02:33	CM S	0.19	132.9
11/2/2018	21:03:05	CM N	0.11	279.3	11/2/2018	22:02:33	CM S	0.04	292.0
11/2/2018	22:03:05	CM N	0.07	88.8	11/2/2018	23:02:33	CM S	0.25	319.2
11/2/2018	23:03:05	CM N	0.22	86.2	12/2/2018	0:02:33	CM S	0.36	321.1
12/2/2018	0:03:05	CM N	0.29	83.5	12/2/2018	1:02:33	CM S	0.46	318.3
12/2/2018	1:03:05	CM N	0.30	80.6	12/2/2018	2:02:33	CM S	0.43	315.8
12/2/2018	2:03:05	CM N	0.30	79.6	12/2/2018	3:02:33	CM S	0.44	331.2
12/2/2018	3:03:05	CM N	0.26	74.4	12/2/2018	4:02:33	CM S	0.42	323.7
12/2/2018	4:03:05	CM N	0.23	73.4	12/2/2018	5:02:33	CM S	0.35	318.6
12/2/2018	5:03:05	CM N	0.20	66.0	12/2/2018	6:02:33	CM S	0.37	314.7
12/2/2018	6:03:05	CM N	0.22	63.8	12/2/2018	7:02:33	CM S	0.38	317.9
12/2/2018	7:03:05	CM N	0.24	63.3	12/2/2018	8:02:33	CM S	0.37	320.4
12/2/2018	8:03:05	CM N	0.26	60.9	12/2/2018	9:02:33	CM S	0.37	320.1
12/2/2018	9:03:05	CM N	0.29	57.6	12/2/2018	10:02:33	CM S	0.29	319.2
12/2/2018	10:03:05	CM N	0.29	61.7	12/2/2018	11:02:33	CM S	0.30	318.6
12/2/2018	11:03:05	CM N	0.28	63.7	12/2/2018	12:02:33	CM S	0.24	319.0
12/2/2018	12:03:05	CM N	0.24	64.5	12/2/2018	13:02:33	CM S	0.14	323.8
12/2/2018	13:03:05	CM N	0.13	67.9	12/2/2018	14:02:33	CM S	0.11	128.0
12/2/2018	14:03:05	CM N	0.12	254.7	12/2/2018	15:02:33	CM S	0.40	133.7
12/2/2018	15:03:05	CM N	0.16	259.0	12/2/2018	16:02:33	CM S	0.61	137.7
12/2/2018	16:03:05	CM N	0.19	257.0	12/2/2018	17:02:33	CM S	0.66	136.7
12/2/2018	17:03:05	CM N	0.19	261.4	12/2/2018	18:02:33	CM S	0.67	136.8
12/2/2018	18:03:05	CM N	0.15	268.1	12/2/2018	19:02:33	CM S	0.63	134.2
					12/2/2018	20:02:33	CM S	0.53	132.4
					12/2/2018	21:02:33	CM S	0.40	132.8
					12/2/2018	22:02:33	CM S	0.24	130.2
					12/2/2018	23:02:33	CM S	0.05	273.0
					13/2/2018	0:02:33	CM S	0.26	320.1
					13/2/2018	1:02:33	CM S	0.35	322.5
					13/2/2018	2:02:33	CM S	0.35	321.4
					13/2/2018	3:02:33	CM S	0.40	319.9
					13/2/2018	4:02:33	CM S	0.37	320.1
					13/2/2018	5:02:33	CM S	0.36	329.3
					13/2/2018	6:02:33	CM S	0.33	325.3
					13/2/2018	7:02:33	CM S	0.37	326.9
					13/2/2018	8:02:33	CM S	0.40	325.0
					Date	Time	ST	CS (m/s)	CD (°)
					13/2/2018	9:02:33	CM S	0.42	324.1
					13/2/2018	10:02:33	CM S	0.41	317.5
St = Station		CS = Current speed				CD = Current direction

**Table 5:** Comparison between mean nutrients value with Malaysia Marine Water Quality Criteria and Standard (MWQCS).

	Class				
Parameter (µg/L)	1	2	3	E	Current study
Ammonia	35	70	320	70	43 (Class 2)
Nitrate	10	60	1000	60	33 (Class 2)
Phosphate	5	75	670	75	6 (Class 2)

Beneficial uses					
Class 1	Preservation, marine protected area, marine parks				
Class 2	Marine life, fisheries, coral reefs, recreational, and mariculture				
Class 3	Ports, oil & gas fields				
Class E	Mangroves, estuarine & river-mouth water				

## 2 Experimental Design, Materials and Methods

### 2.1 Description of the study area

Setiu Lagoon, which is located within Setiu Wetland, is known for its diverse array of biological diversity and has been listed as one of 17 priority conservation sites in the Malaysian Wetland Directory [1]. This 14 km-length lagoon is generally shallow with water depths ranging between 0.3 and 3.2 m, and a tidal range of less than 2 m [2]. The main freshwater sources in Setiu Lagoon come from Setiu River that flows directly into the lagoon and Berambak Lake, which is connected through Ular River (Fig. 1). At the upstream area of Setiu River, there is a presence of an urban area of Penarik Town (Fig. 1). The presence of vegetated sand islands within the lagoon contributed to the narrow feature of this area (Fig. 1). Despite being shallow and narrow, aquaculture and agriculture activities are growing rapidly within the vicinity of Setiu Lagoon (Fig. 1) [1], [2], [3], which introduced pollution that lead to water quality degradation [4], [5].

### 2.2 Experimental design

The research samplings for this data were carried out from 6 to 8 of August 2017, 26 to 28 of December 2017 and 11 to 13 February 2018, which represented the southwest monsoon, wet period of northeast monsoon and dry period of northeast monsoon, respectively. For water quality data, each station was sampled twice per day; once during a low tide and the other during a high tide on the second and third day of the sampling period. The placement of each station was intentionally planned to be located near to the inlet, since one of the motivations of this study is to understand the water renewal time between the lagoon and its adjacent sea. S1 and S2 are located at the downstream of Setiu River, which are exposed to the domestic waste input from Penarik Town. S3 was chose due to its location that is situated at the river mouth area. Meanwhile, S4 to S8 are located near the aquaculture farms and ponds. Since Setiu Lagoon has a shallow water depth, the water quality parameters were sampled at the surface layer of approximately 0.2 m water depth. It is important to note that, there are some missing data from Table 1 was due to difficulty to access the stations because of shallow water depths especially during the low tide. Because there are some missing values, the water quality data was first averaged by the tidal phases; high and low tides, and then averaged by seasons in observing the variability of parameters involved (see reference [1]). For the current meter deployment, CM N is used to represent the current flow in the north of the inlet area, while CM S represents the current flow in the south. The current speed and direction from these two points is very important in representing the water current flow associated with ebb and flood tidal cycles, freshwater discharges and monsoonal forces, which may influence the distribution of nutrients and chlorophyll-*a* in the lagoon.

## 3 Materials and Methods

For the measurement of temperature and salinity, SonTek CastAway Conductivity, Temperature and Depth (CTD) profiler was used according to the measurement time as indicated in Table 3. Water samples for the nutrients and chlorophyll-*a* analysis were filtered through GF/F filter paper (47 mm diameter, nominal pore size 0.7 µm) on-site and stored frozen. Laboratory analyses for nitrate, phosphate and ammonia were conducted by using Westco SmartChem 200 Discrete Analyser with United States Environmental Protection Agency 353.2, 365.1 and 350.1 methods, respectively [6]. Meanwhile, the silicate analysis was carried out according to USGS-I-2700–85 method [7]. Prior to sample analysis, the calibration for the nutrients was performed by preparing the highest concentration of standard. The detection limit was 0.03 µM for phosphate, 0.2 µM for ammonia and nitrate and 0.8 µM for silicate. Concentrations of chlorophyll-*a* were analysed in the laboratory with an adopted procedure from American Public Health Association, 2005 method [8] using a UV–VIS Spectrophotometer. Two units of Valeport Current Meters were also moored on 6 to 8 August 2017, 26 to 28 of December 2017 and 11 to 13 February 2018 at CM N and CM S to obtain the temporal variability of the current flow in the lagoon. Both current meters were programmed to record the data with 60 min interval.

## Declaration of Competing Interest

The authors declare that they have no known competing financial interests or personal relationships which have, or could be perceived to have, influenced the work reported in this article.

## Appendix Supplementary materials

Image, application 1

Image, application 2
