# Supplementary material for: Water quality data in a shallow and narrow Setiu Lagoon
Source: Data Brief. 2021 Feb 11;35:106866. doi: 10.1016/j.dib.2021.106866 (PMC8010623; doi:10.1016/j.dib.2021.106866)
Supplement: Supplementary file 1 [file mmc1.pdf]

CREDIT AUTHOR STATEMENT

Manuscript title:

Water quality data in a shallow and narrow Setiu Lagoon

All persons who meet authorship criteria are listed as authors, and all authors certify that they have participated sufficiently in the work to take public responsibility for the content, including participation in the concept, design, analysis, writing, or revision of the manuscript. Furthermore, each author certifies that this material or similar material has not been and will not be submitted to or published in any other publication before its appearance in the journal of *Data in Brief*.

Authorship contributions

Zuraini Zainol: Conceptualization, Methodology, Formal Analysis, Investigation, Writing - Original Draft

Mohd Fadzil Akhir: Conceptualization, Methodology, Writing - Review & Editing, Supervision

Afifi Johari: Methodology, Investigation

Azizi Ali: Methodology, Investigation

Acknowledgements
All persons who have made substantial contributions to the work reported in the manuscript (e.g., technical help, writing and editing assistance, general support), but who do not meet the criteria for authorship, are named in the Acknowledgements and have given us their written permission to be named. If we have not included an Acknowledgements, then that indicates that we have not received substantial contributions from non-authors.
