# Supplementary material for: Water quality data in a shallow and narrow Setiu Lagoon
Source: Data Brief. 2021 Feb 11;35:106866. doi: 10.1016/j.dib.2021.106866 (PMC8010623; doi:10.1016/j.dib.2021.106866)
Supplement: Supplementary file 2 [file mmc2.pdf]

Declaration of Interest Statement

Manuscript title: Water quality data in a shallow and narrow Setiu Lagoon

The authors whose names are listed immediately below certify that they have NO affiliations with or involvement in any organization or entity with any financial interest (such as honoraria; educational grants; participation in speakers' bureaus; membership, employment, consultancies, stock ownership, or other equity interest; and expert testimony or patent-licensing arrangements), or non-financial interest (such as personal or professional relationships, affiliations, knowledge or beliefs) in the subject matter or materials discussed in this manuscript.

Author names:

**Zuraini Zainol**	**6 December 2020**
**Mohd Fadzil Akhir**	**6 December 2020**
**Afifi Johari**	**6 December 2020**
**Azizi Ali**	**6 December 2020**
